# Pedican: an online gene resource for pediatric cancers with literature evidence

**DOI:** 10.1038/srep11435

**Published:** 2015-06-15

**Authors:** Min Zhao, Lei Ma, Yining Liu, Hong Qu

**Affiliations:** 1 Center for Bioinformatics, State Key Laboratory of Protein and Plant Gene Research, College of Life Sciences, Peking University, Beijing 100871, P.R. China

## Abstract

Pediatric cancer (PC), that is cancer occurring in children, is the leading cause of death among children worldwide, with an incidence of 175,000 per year. Elucidating the genetic abnormalities and underlying cellular mechanisms may provide less toxic curative treatments. Therefore, it is important to understand the pathology of pediatric cancer at the genetic, genomic and epigenetic level. To unveil the cellular complexity of PC, we have developed a database of pediatric cancers (Pedican), the first literature-based pediatric gene data resource by comprehensive literature curation and data integration. In the current release, Pedican contains 735 human genes, 88 gene fusion and 24 chromosome abnormal events curated from 2245 PubMed abstracts. Pedican provides detailed annotations for each gene, such as Entrez gene information, involved pathways, protein–protein interactions, mutations, gene expression, methylation sites, TF regulation, and post-translational modification. Additionally Pedican has a user-friendly web interface, which allows sophisticated text query, sequence searches, and browsing by highlighted literature evidence and hundreds of cancer types. Overall, our curated pediatric cancer-related gene list maps the genomic and cellular landscape for various pediatric cancers, providing a valuable resource for further experiment design. The Pedican is available at http://pedican.bioinfo-minzhao.org/.

Pediatric cancer (PC) is the second leading cause of death among children of 5~14 years of age in the United States, trailing only behind fatal accidents^1^. It is also estimated that 175,000 cases per year of children (less than 15 years old) were diagnosed with cancer worldwide^1^. Less than 40% of patients (those mainly from high-income countries) are able to receive adequate treatment^2^,^3^. In addition, children with cancer are at high risk of mental problems. Though the survival rate of PC has continuously improved by the use of radiotherapy and chemotherapy, the adverse effects may substantially affect the quality of life for survivors^4^,^5^. Elucidating the genetic abnormalities and underlying cellular mechanisms which initiate the cancer may provide earlier diagnosis and less toxic treatments. Therefore, it is important to understand the pathology of pediatric cancer at the genetic, genomic and epigenetic levels.

The pioneer effort in Surveillance, Epidemiology, and End Results (SEER) program from the National Cancer Institute (NCI) was to collect PC patients’ medical records, including the incidence of childhood cancer in the United States, began in 1975, gathering large amounts of information on survival, gender differences, and geographical distribution^6^. The accumulated single gene-based association studies showed that PCs are distinct from adult cancers^4^. Recently, population-based genetic screening was initiated by St. Jude Children’s Hospital and the University of Washington Children’s Cancer Genome Project (The Pediatric Cancer Genome Project, PCGP) in 2010^7^. As the world’s largest genetic analysis of PC, PCGP created the first genetic landscape of 15 major PCs by next-generation sequencing at a cost of about $ 65 million.

However, the PCGP focused on the major PC types. The official PCGP website provides PCGP data, not containing the information from published literature. Another pediatric related web resource, pond4kids, is made up of hospital-based cancer registration and clinical information, not including patient genetic data. The genetic abnormality relating to other harmful PCs are scattered in the literature without systematic collection and comparison. In this study, we integrated known genetic predisposition information from thousands of cases in the literature to complement the population-based study from PCGP. To this aim, 2245 PC-related PubMed abstracts were collected and manually curated, which result in 735 human PC-related human genes, 88 gene fusion events, and 24 chromosome-level events being recorded. Moreover, we provide comprehensive biological annotation for biological pathway, gene regulation, interaction and expression in a user-friendly way, which may help the PC community to obtain a better understanding of pathogenesis for various PCs, and even facilitate the gene prioritization and prediction for PCs. In addition, this data resource also makes it feasible to compares the genetic differences for the cancers in children and adults.

## Results

### Functional enrichment analyses pinpoint development-related NOTCH1, FGFR and GAB1 signaling transduction in PC

To explore the relevant biological processes of our collected genes, gene-set enrichment analysis was adopted to characterize whether the 735 PC-related genes had any significant annotations comparing to all the human protein-coding genes. Using strict cutoff (corrected p-value less than 0.01 and the annotated genes more than 30% of all PC-related genes); we identified 35 statistically significant enriched pathways (Table S1) and 170 gene ontology terms (Table S2). Those enriched functional pathways are mainly related to cancers such as transcriptional mis-regulation, constitutive PI3K/AKT signaling, proteoglycans and the P53 signaling pathway (Table 1). Notably, the top enriched gene ontology terms are all related to development processes, such as cell fate commitment, gland development, regulation of organ morphogenesis, stem cell proliferation, mesenchyme development, and morphogenesis of a branching epithelium.

In fact, the pathway analysis result also confirmed the gene ontology result. The PC-related genes were also highly enriched in development-signaling pathways such as Notch1 intracellular domain regulates transcription, constitutive signaling by Notch PEST domain mutants, downstream signaling of activated FGFR, and the GAB1 signalosome. The Notch signaling pathway has a dual role in cancer (oncogenic and tumor suppressor functions)^8^. It is hypothesized that Notch tends to modulate the epithelial mesenchymal transition (EMT) during cancer metastasis^9^. However, the role of Notch signaling in PCs has only been studied in childhood T cell acute lymphoblastic leukemia (T-ALL)^10^. More extensive studies of Notch signaling in other PCs will provide a rationale for Notch-based therapeutic strategies. FGFR is the receptor for fibroblast growth factors (FGFs), which are often relevant to cell stemness, proliferation, anti-apoptosis, drug resistance, and angiogenesis^11^. In our Pedican, four FGFRs (FGFR1, FGFR2, FGFR3, FGFR4) were recorded to be related to PCs. For example, FGFR1 was reported to be associated with tumorigenesis of Ewing’s sarcoma^12^ and Rhabdomyosarcoma^13^. It was demonstrated that FGFR inhibitors have an effect on overcoming drug resistance, thus FGFR-based therapeutic strategy is promising. More systematic studies using a targeted-sequencing approach will be useful to detect more candidate mutations in other PCs. GAB1 is a docking protein to transduce cellular signals from tyrosine kinases, such as Met (the hepatocyte growth factor) and EGFR (the epidermal growth factor receptor). The role of GAB1 signalosome in cancer was only reported in breast^14^ and colorectal cancers^15^. Though GAB1 is not included in our Pedican as there is not direct link of GAB1 to any PCs, the other components of the GAB1 signalosome are enriched in our 735 PC-related genes, such as PDGFB, PDGFA, EGFR, MDM2, CDK4, PDGFRA. In summary, our results highlight that multiple cellular signaling events are related to PCs, especially NOTCH, FGFR and GAB1 signaling. The GAB1 is a good candidate gene to test its functions in PCs and other adult cancers.

### PC-related genes are enriched in adult cancers, preterm birth and high birth weight

Though previous studies show that the PCs are different from their corresponding adult cancers^4^, our disease-based enrichment analysis still shows connections between the PC-related genes and a broad-spectrum of human adult cancers (Table S3). Even the enrichment analysis of PC-related genes cannot measure how much commonality exists for the underlying molecular mechanisms between PCs and adult cancers; instead, it may imply that the overall signaling pathways of PCs are similar to adult cancers. The cancers involved mainly include those of the breast, colorectal, lung, stomach, esophageal, leukemia, bladder, prostate, pancreas, cervix, liver, melanoma, ovary and glioma. Systematic comparison of PCs with adult cancers may provide more comprehensive picture for the underlying common molecular mechanism between PCs and adult cancers.

Most interestingly, the 735 PC-related genes are also over-represented in endometriosis, type 1 diabetes (T1D), benzene toxicity, primary biliary cirrhosis, preterm birth, and high birth weight. The positive association of high birth weight to both childhood and adult cancers is shown by several studies^16^,^17^,^18^,^19^,^20^,^21^. Though the risk of preterm birth to an increased incidence of breast cancer in the mother has been discussed previously^22^,^23^, there is no direct evidence linking preterm birth to PCs. Our enrichment analysis may provide a clue for further exploration on the potential role of preterm birth in PCs. Therefore, further data mining on our Pedican may provide a clue about a potential role of birth weight and preterm birth in both PCs and adult cancers, including changes of hormone signaling along the cancer development.

### Prioritize the key genes in PC and their mutational landscape in pan-cancer genomic data

To systematically evaluate the importance of PC-related genes, we conducted a gene ranking using 47 reliable genes as a training set by Endeavour (see Methods). The top ten ranked genes, included CDK4, CCND2, IGF1R, PDGFRB, CHEK2, CASP10, ERBB3, ATR, and E2F1. Not surprisingly, the majority of these top ranked genes are involved in the key pathway of cancers such as the cell cycle and P53 signaling pathway.

**Figure 1:**
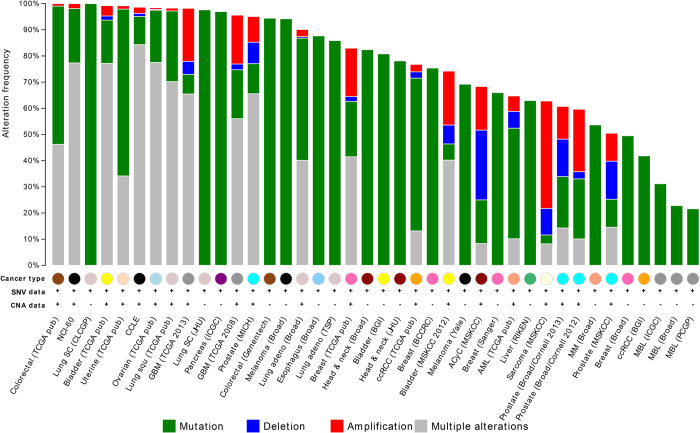
The mutational landscape for the top 100 PC-related genes in multiple cancers.

Although our collected genes have been demonstrated to have abnormal gene expression or other functional relevance to PCs, the systematic examination of the genetic variants in pan-cancer has not yet been conducted. These mutational patterns are useful for comparing the PCs with their counterpart adult cancers. As shown in Fig. 1, the top 100 ranking PC-related genes (including 47 genes from the training set and 53 top ranked genes from Endeavor) have overwhelming mutations in adult cancers. It is interesting that the 100 genes are over 90% mutated in a few cancers and cell lines including colorectal cancer, lung small cell cancer, bladder cancer, uterine cancer, ovarian cancer, squamous cell lung cancer, glioblastoma multiforme, pancreatic cancer, prostate cancer and melanoma. This result may highlight that PCs share substantial molecular mechanisms from adult cancers. The further comparison between specific PC and its corresponding adult cancer may provide more clues.

### The PC-related protein-protein interaction network is highly modularized

**Figure 2:**
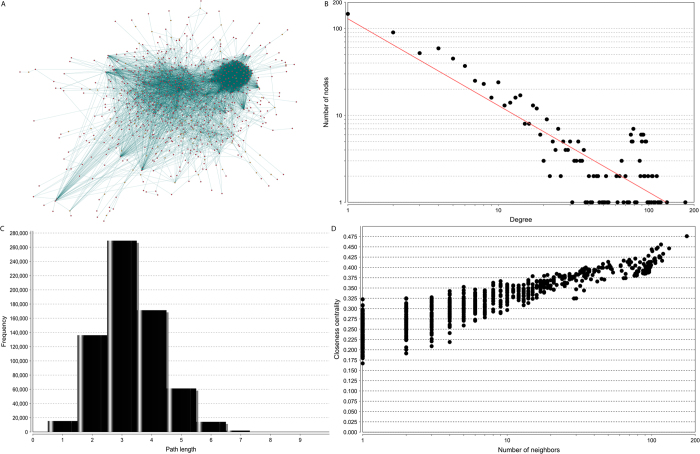
Reconstructed PC map using protein-protein interaction data. (**A**) The 335 genes in red are genes from the core dataset in our Pedican. The remaining 36 genes in orange are linker genes that bridge the 335 genes; (**B**) the degree distribution; (**C**) the short path length frequency; (**D**) the correlation between closeness centrality and the number of neighbours.

By using the integrative protein-protein interaction data from the Pathway Commons database^24^, we performed a pathway reconstruction to present a cellular map related to PC. The reconstructed PC-related protein-protein interaction network contains 819 genes and 7720 gene-gene interactions with existent evidence from known biological pathways (Fig. 2). Among the 819 nodes, 725 are from our curated 735 PC-related genes. The remaining 94 are the linker genes to bridge the PC-related genes to form a fully connected map. Therefore, the majority of curated PC-related genes are organized in a highly modular structure. This is not only supportive of the precision of our data curation, but it also reveals the PC-related genes are acting in a high-density cellular module.

### The common cancer genes across multiple PC types

**Figure 3:**
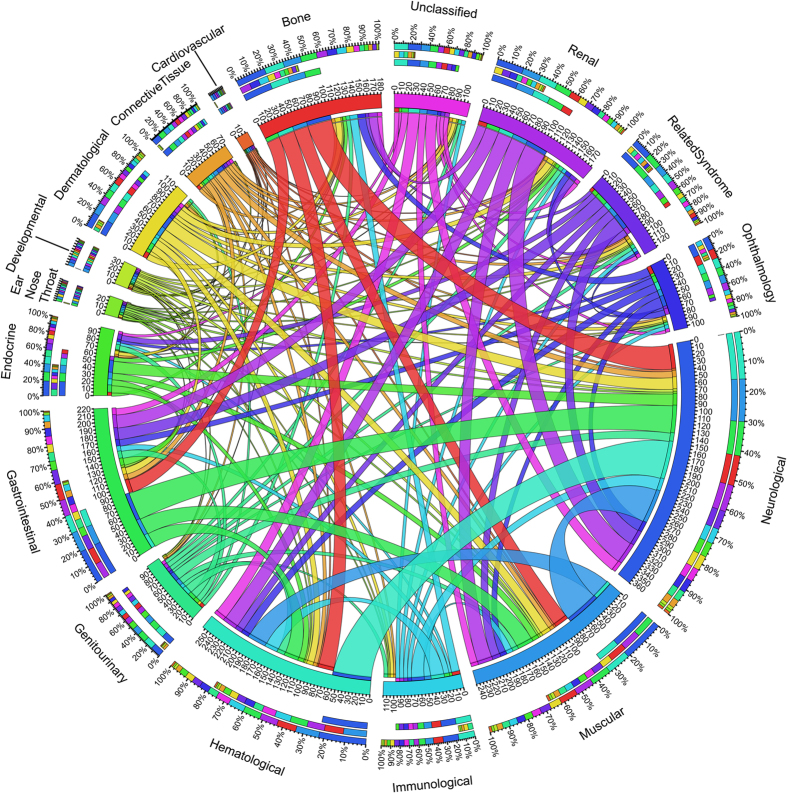
The shared genes across multiple PCs. The length of circularly arranged segments is proportional to the total genes in each PC group. The ribbons connecting different segments represent the number of shared genes between PC groups. The three outer rings are stacked bar plots that represent relative contribution of other PC group to the PC group totals.

On the basis of information from the literature, we annotated all the genes in Pedican with a specific cancer type. We classified all the PC types into 17 major groups according to anatomic and biological functions, including bone, cardiovascular, connective tissue, dermatological, developmental, ear/nose/throat, endocrine, gastrointestinal, genitourinary, hematological, immunological, muscular, neurological, ophthalmology, related syndrome, renal, and unclassified. The majority of PC cancer-related genes are related to neurological (357) and blood (220) functions. Based on the common genes in the 17 PC groups, the overlapping relationships were plotted in Fig. 3. It revealed that the multiple cancer groups shared potential molecular mechanisms. For instance, 58 common genes are found between neurological-related cancers and haematological-related cancers.

## Conclusion

Pedican is constructed as a free database and analysis server to enable users to rapidly search and retrieve summarized PC-related genes. The functional enrichment analyses reveal that multiple developmental processes are related to PC-related genes involved in various cancer types. Our curated gene list provides a clue to the discovery of the common driver genes across multiple PCs and to explore the difference between the adult cancers and their counterpart PCs. The Pedican is freely accessible at http://Pedican.bioinfo-minzhao.org/.

### Limitations and future work

This study aims to integrate literature and genomic data to explore the common mechanisms for different pediatric cancers. Comparing with the other public databases, our pediatric cancer database provided a curated, organized, and annotated gene list for pediatric cancer in an easily accessible way. From our web interface, user can not only find the reported genes related to pediatric cancer with their origin references, but also obtain more comprehensive knowledge about these collected genes. This culmination of long-standing and high quality resource in pediatric cancer may address the genetic improvement of pediatric cancer treatment. For example, our reconstructed PC protein-protein interaction network may help researchers to connect novel candidates to the known pediatric cancer genes and sum up the small genetic effects in biological pathway and network level. Additionally, our systematic comparison to explore the common pediatric-related genes may be useful for further experimental design of genetic screen for different PCs. However, the further data integration in multiple dimensions may provide deeper insight about the common and uniqueness of pathogen between pediatric cancers and adult cancers. Comparing to adult cancer project from TCGA, the PCGP project does not provide a comprehensive genomics features such as methylation, microRNA regulation, lncRNA expression. This may limit our understanding of differences between pediatric and corresponding adult cancer precisely.

Armed with the knowledge in pediatric cancer databases and other members of pediatric cancer data, we will establish genomics-informed programs that will improve our understanding of pediatric cancers. In current study, we built the database based on literature curation, which may slow down our database update cycle. Due to the difficulty to collect pediatric cancer samples, the small scale studies related to PCs are not increasing dramatically. To obtain update of relevant literature, we constructed automatic literature searching terms using My NCBI tool, which will return matched published articles every two weeks. According to the statistics during the past half year, we can only receive about 0–10 abstracts from PubMed using our search expression in the manuscript. We may consider to use the literature similarity to cluster the newly available literature to accelerate our curation. Another limitation for our database may arise from the bioinformatic annotation since the fast development in the cancer genomics field. To address this potential problem, we have implemented an automatic system to import functional information from a variety of data sources, which can help us integrate more genes with relevant annotations. Once the data content update, the web interface will be updated accordingly annually.

## Methods

### Extensive literature search for PC-related genes and gene curation

**Figure 4:**
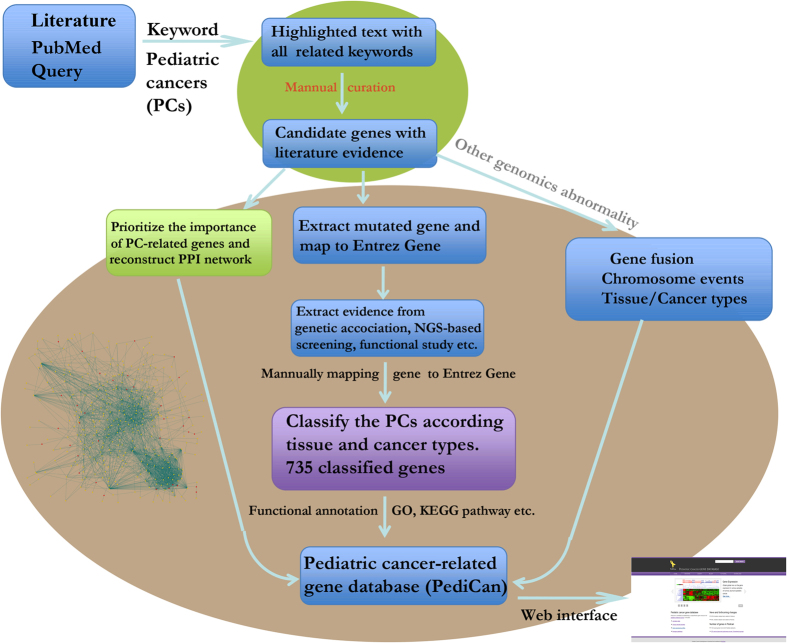
Pipeline for collection, expansion and annotation of PC-related genes.

To build our comprehensive collection of PC-related genes, we conducted an extensive literature search and curation in four steps (Fig. 4): (i) literature searching against PubMed (on Oct 20th, 2013) using complex expression: (“pediatric”[Title/Abstract] OR “child”[Title/Abstract] OR “childhood”[Title/Abstract]) and (“cancer”[Title/Abstract] OR “tumor”[Title/Abstract] OR “carcinoma”[Title/Abstract]) AND ((“genome-wide association study” [Title/Abstract] OR “genome wide association study” [Title/Abstract]) OR (“gene”[Title/Abstract] AND (“association”[Title/Abstract] OR “microarray” [Title/Abstract] OR “expression” [Title/Abstract] OR “linkage” [Title/Abstract] OR “proteomics” [Title/Abstract] OR “genetic” [Title/Abstract] OR “metabolomics” [Title/Abstract] OR “copy number variation” [Title/Abstract] OR “idiopathic” [Title/Abstract] OR “hereditable” [Title/Abstract] OR “family” [Title/Abstract] OR “mouse model” [Title/Abstract] OR “animal model” [Title/Abstract] OR “microRNA” [Title/Abstract] OR “mutation” [Title/Abstract] OR “SNP” [Title/Abstract] OR “drug” [Title/Abstract] OR “transporter” [Title/Abstract]))); (ii) retrieving all the resulting 2245 abstracts and grouping the literature using the “Related Articles” function in Entrez system; (iii) extracting PC-related text from the grouped articles. Those sentences related to PC were manually curated to obtain the correct gene names and cancer types; (iv) the extracted candidate gene name and alias were mapped to NCBI Entrez gene database manually. As a result, the 735 Entrez human Gene IDs with high confidence were collected as PC-related genes. In addition to collecting the mutated genes, the gene fusion events and other chromosome events were also curated. Based on the curated cancer types, we grouped all the PC types into 17 major groups according anatomic and biological functions. The overlapping cancer genes across cancer types were visualized using Circos^25^.

### Biological functional annotations and database construction

To characterize the meaningful biological function, we retrieved comprehensive functional information from public resources, including crosslinks to NCBI Entrez gene^26^, UniProt^27^, Ensembl^28^, Gene Ontology^29^, transporter substrate database^30^, BioCyc^31^, KEGG Pathway^32^, rate-limiting enzyme database^33^ PANTHER^34^, PID Curated^35^, pathway localization database^36^ and PID Reactome^37^,^38^. The relevant disease information was collected from GAD (gene association database)^39^, KEGG Disease^40^, Fundo^41^,^42^, NHGIR^43^, as well as OMIM^26^. The comprehensive mRNA expression profiling data from both normal and tumor tissues was incorporated from the BioGPS database^44^. Moreover, the original PC related articles in the NCBI PubMed database are hyperlinked to each gene. Additionally, we also collected the mutation information from the PCGP^7^ and COSMIC^45^ databases. The protein-protein interaction data were integrated from the pathway commons database. To help construct regulatory networks, we also obtained various upstream and downstream regulators in humans with emphasis on their regulatory transcription factors.

### Gene ranking using Endeavour and cancer mutational pattern in multiple cancer types

Though we collected hundreds of genes related to various PC types, the common driver genes are still unclear because of its high genetic heterogeneity. All the PC-related genes are scattered in individual studies, which often focus on verifying specific genes/variants predisposing to PCs. Thus, data integration and evaluation across all the cancer types may help to highlight some important common driver mutated genes. To this goal, we adopted the Endeavour gene ranking tool^46^ to prioritize all the 735 genes in the Pedican. Basically, the Endeavour extract features on the training gene list by using a multiple dimensional dataset, including gene expression, protein-protein interaction information, biological annotations, sequence features, and literature evidences. Here, we extracted a well-known PC-related list include 47 genes (TP53, WT1, MYCN, RB1, SMARCB1, CDKN2A, MYC, RET, MDM2, ABCB1, IGF2, NF1, PMS2, CTNNB1, BRAF, ALK, PTCH1, MLH1, CDKN1A, BCL2, APC, MSH2, MLL, EGFR, CDKN1B, NTRK1, RASSF1, PTEN, MSH6, CDKN2B, CCND1, WNT1, SHH, PDGFRA, VHL, KRAS, CD99, CASP8, ATM, TNF, MTHFR, ETV6, CDKN1C, TFE3, NBN, KIT, and ERBB2) with at least 10 abstract evidences to build a ranking model. Using all the remaining 688 genes as input, Endeavour starts to prioritize the genes using multiple extracted features. As a result, the Endeavour system integrates all the outputs from the training models to form a global ranking for all the candidate PC-related genes by order statistics. Totally, there are 643 human genes which were ranked (Table S4). The top ten ranked genes include CDK4, CCND2, IGF1R, PDGFRB, CHEK2, CASP10, ERBB3, ATR, E2F1, and RBL2 (Table 2). Not surprisingly, majority of these top ranked genes are involved in key pathways of cancers such as “TGF-beta signaling pathway”. Although these candidate genes have been demonstrated to have abnormal gene expression or other functional relevance to some PCs, most of them are not detected as genetic variants in all the reported PCs, which are useful for users to screen potential genes for new PCs and other diseases (Table S4). The top 100 ranking of PC related-genes are inputted into the cBio portal to obtain a mutation pattern across multiple cancers^47^.

### Reconstructing a protein-protein interaction network related to PC genes

To explore the frequently mutated genes in PCs and determine the underlying biological mechanisms, we build a protein-protein interacting map based on all the 735 PC-related genes. To this aim, we used a non-redundant human interactome from Pathway Commons, containing 3629 nodes and 36,034 protein–protein links. It is noteworthy that the protein-protein interactions are based on pathway databases such as Reactome^37^, which have biological meanings. The final interactome contains pathway-based gene-gene interaction links. To build a sub-network related to the 735 PC-related genes of interest, we used the similar strategies implemented in our previous study^48^. In this approach, all the inputted seed genes were mapped to the human interactome, which was used to produce a sub-network connected with inputted genes, where possible by the shortest paths. The final network visualization and topological properties were generated by using Cytoscape (version 2.8)^49^.

The structure of biological networks is often related to its functions^50^. Generally, the network follows a few topological rules, which are useful for characterizing the potential function. To explore the function of our reconstructed interactome based on PC-related genes, topological analyses were conducted using the NetworkAnalyzer plugin in Cytoscape (Fig. 3b–d)^49^. In this study, the number of connections for each node was defined as degree in a network^50^. To present the shortest steps for one node to reach another, we obtained closeness centrality for each node in the network^50^. The visualization of the whole network was performed by using Cytoscape^49^.

### Pathway enrichment analysis

Throughout the paper, the representative pathways from KEGG and Reactome for each gene set were identified by KOBAS^51^. In these pathway analyses, all the human protein-coding genes were set as background to calculate statistical significance. In addition, the Benjamini-Hochberg multiple testing corrected P-values for enriched pathways were adopted based on hypergeometric test by using KOBAS. Finally, the enriched human pathways with corrected P-values less than 0.01 were identified as over representative pathways for each gene set.

### Web interface development

**Figure 5:**
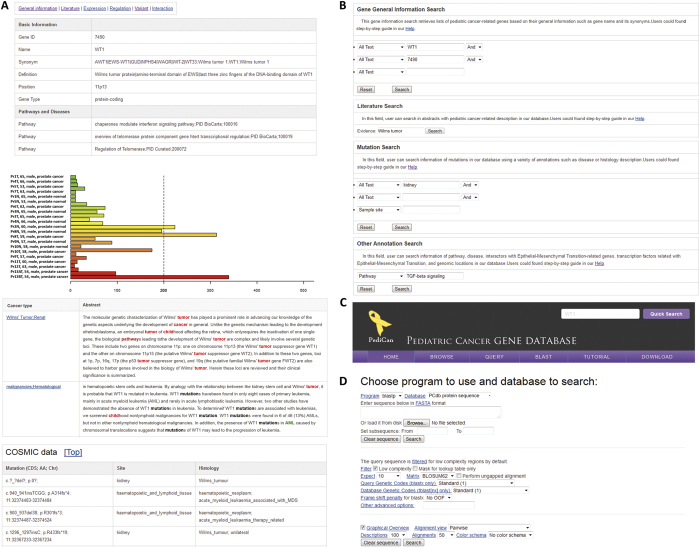
Web interface of Pedican. (**A**) The basic information in each PC-related gene page; (**B**) Query interface for text search; (**C**) BLAST search interface for comparing query against all sequences in Pedican; (**D**) Browser interface for genes in top 10 enriched pathways, top 10 enriched diseases and shared cytobands.

To provide a web interface for the public to access our collected information, we stored all the data and annotations in the relational database management system MySQL, which is open source and reliable. Using the Perl CGI module and JavaScript technology, a web interface was implemented to read and browse the database. The apache web server on a Linux server was used to publish the web pages. In Pedican, all the human genes are mapped to NCBI Entrez gene IDs, which is able to comprehensively hyperlink to various bioinformatics data resources (Fig. 5A). For all the genes in Pedican, we provided five sub-pages to characterize five annotation categories, including the general gene sequence information, the literature evidence to PC, the upstream regulators and other regulation events, the mutational pattern in PCGP data and COSMIC database, and the protein-protein interaction data. For example, by incorporating the gene expression profile from BioGPS, we generated an expression bar chart to present an overview for various normal tissues and cancer individuals (Fig. 5A). For our curated literature related to each gene, we highlighted their abstracts with keywords related to cancer or pediatric disorder for the users to review them conveniently.

To help user to do text query against our Pedican data, we developed six powerful query forms regarding pathway and disease information, genomic location, literature evidence, and gene expression range in normal/cancer samples, and mutation information (Fig. 5B). Notably, a quick text search for GeneID, gene symbol, and gene alias is on the top right of each page, which is useful for the user to retrieve any data in the database quickly. In addition, users can also run a sequence similarity search (BLAST) against the nucleotide and protein sequences in Pedican (Fig. 5C). Users can also explore the data in Pedican using a web browser; including the PC type, the organ/tissue classification, significantly enriched pathway, related disease, reported linkage region, and chromosome number (Fig. 5D). For each related KEGG pathway, the marked chart is provided to highlight the entire related PC-related genes. Finally, for the purpose of offline data usage, we provide a downloadable gene list corresponding to all the PC types in a plain text for all the collected 735 genes related to PC.

## Additional Information

**How to cite this article**: Zhao, M. *et al*. PediCan: an online gene resource for pediatric cancers with literature evidence. *Sci. Rep*. **5**, 11435; doi: 10.1038/srep11435 (2015).

## Supplementary Material

Supplementary Table S1

Supplementary Table S2

Supplementary Table S3

Supplementary Table S4

## Figures and Tables

**Table 1:** The statistically significant enriched pathways of PC-related genes.

*Pathway*	Adjusted P-values*
**KEGG pathway**	
Pathways in cancer	7.78E-15
Bladder cancer	4.22E-07
Transcriptional misregulation in cancer	1.23E-06
Melanoma	1.56E-06
Prostate cancer	3.67E-06
Colorectal cancer	2.03E-05
Chronic myeloid leukemia	2.16E-05
Hepatitis B	4.00E-05
Proteoglycans in cancer	6.62E-05
Endometrial cancer	0.00022523
Glioma	0.000346597
Pancreatic cancer	0.000416823
Thyroid cancer	0.000563627
Non-small cell lung cancer	0.000715898
Renal cell carcinoma	0.001631639
p53 signaling pathway	0.002305302
Acute myeloid leukemia	0.004965593
**Reactome pathway**	
Constitutive PI3K/AKT Signaling in Cancer	5.27E-06
PI3K/AKT activation	1.11E-05
Signaling by SCF-KIT	1.34E-05
Signaling by FGFR	1.91E-05
PI-3K cascade	2.04E-05
PIP3 activates AKT signaling	2.04E-05
PI3K events in ERBB2 signaling	2.04E-05
PI3K/AKT Signaling in Cancer	2.04E-05
PI3K events in ERBB4 signaling	2.04E-05
Downstream signaling of activated FGFR	2.10E-05
Signaling by ERBB4	2.14E-05
GAB1 signalosome	3.45E-05
Role of LAT2/NTAL/LAB on calcium mobilization	4.12E-05
Downstream signal transduction	4.55E-05
NOTCH1 Intracellular Domain Regulates Transcription	0.000662652
Constitutive Signaling by NOTCH1 HD+PEST Domain Mutants	0.001169199
Constitutive Signaling by NOTCH1 PEST Domain Mutants	0.001169199

Note: *Adjusted *P*-values: the *P*-values of the hypergeometric test were corrected by Benjamini-Hochberg multiple testing correction.

**Table 2:** The top 30 ranked PC-related genes.

*GeneSymbol (Ranked)*	*Global Rank P-value*^*1*^	*Global Rank ratio*^*2*^
CDK4	2.59E-07	0.00156
CCND2	2.34E-06	0.00311
IGF1R	2.64E-06	0.00467
PDGFRB	6.04E-06	0.00622
CHEK2	1.17E-05	0.00778
CASP10	3.87E-05	0.00933
ERBB3	6.77E-05	0.0109
ATR	0.000087	0.0124
E2F1	0.000119	0.014
RBL2	0.000148	0.0156
CDK2	0.000158	0.0171
EP300	0.000186	0.0187
ABL1	0.000238	0.0202
TP73	0.000238	0.0218
TSC2	0.000297	0.0233
RAF1	0.000313	0.0249
CREBBP	0.000386	0.0264
MAP2K1	0.000424	0.028
AKT1	0.000432	0.0295
HRAS	0.00044	0.0311
AXIN1	0.000447	0.0327
BAX	0.00045	0.0342
BRCA1	0.000483	0.0358

Note: ^1^ represent the probability that a candidate gene would obtain these ranks by chance. ^2^ the global ranking ratio from Endeavour.

## References

[b1] Ward E., Desantis C., Robbins A., Kohler B. & Jemal A. Childhood and adolescent cancer statistics, 2014. CA Cancer J Clin 64, 83–103 (2014). 24488779 10.3322/caac.21219

[b2] Kruger M. . Childhood cancer in Africa. Pediatr Blood Cancer 61, 587–592 (2014). 24214130 10.1002/pbc.24845

[b3] Gatta G. . Childhood cancer survival in Europe 1999-2007: results of EUROCARE-5--a population-based study. Lancet Oncol 15, 35–47 (2014). 24314616 10.1016/S1470-2045(13)70548-5

[b4] Stiller C. A. Epidemiology and genetics of childhood cancer. Oncogene 23, 6429–6444 (2004). 15322515 10.1038/sj.onc.1207717

[b5] Chemaitilly W. & Meacham L. R. Epidemiology: Endocrine disorders in adult survivors of childhood cancer. Nat Rev Endocrinol 10, 320–1 (2014). 24709656 10.1038/nrendo.2014.50

[b6] A. L. P.-L., CH J., ME R., M A., DR L. & S P. Revising the Multiple Primary and Histology Coding Rules. Journal of Registry Management 34, 81–86 (2007).

[b7] Downing J. R. . The Pediatric Cancer Genome Project. Nat Genet 44, 619–622 (2012). 22641210 10.1038/ng.2287 PMC3619412

[b8] Lobry C., Oh P. & Aifantis I. Oncogenic and tumor suppressor functions of Notch in cancer: it’s NOTCH what you think. J Exp Med 208, 1931–1935 (2011). 21948802 10.1084/jem.20111855 PMC3182047

[b9] Allenspach E. J., Maillard I., Aster J. C. & Pear W. S. Notch signaling in cancer. Cancer Biol Ther 1, 466–476 (2002). 12496471 10.4161/cbt.1.5.159

[b10] Malyukova A. . The tumor suppressor gene hCDC4 is frequently mutated in human T-cell acute lymphoblastic leukemia with functional consequences for Notch signaling. Cancer Res 67, 5611–5616 (2007). 17575125 10.1158/0008-5472.CAN-06-4381

[b11] Katoh M. & Nakagama H. FGF receptors: cancer biology and therapeutics. Med Res Rev 34, 280–300 (2014). 23696246 10.1002/med.21288

[b12] Schaefer K. L. . Characterization of the malignant melanoma of soft-parts cell line GG-62 by expression analysis using DNA microarrays. Virchows Arch 440, 476–484 (2002). 12021921 10.1007/s00428-001-0558-9

[b13] Goldstein M., Meller I. & Orr-Urtreger A. FGFR1 over-expression in primary rhabdomyosarcoma tumors is associated with hypomethylation of a 5’ CpG island and abnormal expression of the AKT1, NOG, and BMP4 genes. Genes Chromosomes Cancer 46, 1028–1038 (2007). 17696196 10.1002/gcc.20489

[b14] Ortiz-Padilla C. . Functional characterization of cancer-associated Gab1 mutations. Oncogene 32, 2696–2702 (2013). 22751113 10.1038/onc.2012.271

[b15] Seiden-Long I. . Gab1 but not Grb2 mediates tumor progression in Met overexpressing colorectal cancer cells. Carcinogenesis 29, 647–655 (2008). 18192688 10.1093/carcin/bgn009

[b16] Ross J. A. High birthweight and cancer: evidence and implications. Cancer Epidemiol Biomarkers Prev 15, 1–2 (2006). 16434576 10.1158/1055-9965.EPI-05-0923

[b17] Ahlgren M. . Birth weight and risk of breast cancer in a cohort of 106,504 women. Int J Cancer 107, 997–1000 (2003). 14601061 10.1002/ijc.11481

[b18] Mellemkjaer L. . Birth weight and risk of early-onset breast cancer (Denmark). Cancer Causes Control 14, 61–64 (2003). 12708726 10.1023/a:1022570305704

[b19] Vatten L. J. . Birth weight as a predictor of breast cancer: a case-control study in Norway. Br J Cancer 86, 89–91 (2002). 11857017 10.1038/sj.bjc.6600011 PMC2746526

[b20] Lahmann P. H. . Birth weight is associated with postmenopausal breast cancer risk in Swedish women. Br J Cancer 91, 1666–1668 (2004). 15477861 10.1038/sj.bjc.6602203 PMC2409953

[b21] Kaijser M., Akre O., Cnattingius S. & Ekbom A. Preterm birth, birth weight, and subsequent risk of female breast cancer. Br J Cancer 89, 1664–1666 (2003). 14583767 10.1038/sj.bjc.6601357 PMC2394405

[b22] Rooney B. Delayed birth equals more cancers and preterm births. West J Med 174, 385–386 (2001). 11381000 10.1136/ewjm.174.6.385 PMC1071422

[b23] Melbye M., Wohlfahrt J., Andersen A. M., Westergaard T. & Andersen P. K. Preterm delivery and risk of breast cancer. Br J Cancer 80, 609–613 (1999). 10408874 10.1038/sj.bjc.6690399 PMC2362328

[b24] Cerami E. G. . Pathway Commons, a web resource for biological pathway data. Nucleic Acids Res 39, D685–690 (2011). 21071392 10.1093/nar/gkq1039 PMC3013659

[b25] Krzywinski M. . Circos: an information aesthetic for comparative genomics. Genome Res 19, 1639–1645 (2009). 19541911 10.1101/gr.092759.109 PMC2752132

[b26] Sayers E. W. . Database resources of the National Center for Biotechnology Information. Nucleic Acids Res 39, D38–51 (2011). 21097890 10.1093/nar/gkq1172 PMC3013733

[b27] Magrane M. Consortium U. UniProt Knowledgebase: a hub of integrated protein data. Database (Oxford) 2011, bar009 (2011). 21447597 10.1093/database/bar009 PMC3070428

[b28] Flicek P. . Ensembl 2011. Nucleic Acids Research 39, D800–806 (2011). 21045057 10.1093/nar/gkq1064 PMC3013672

[b29] The Gene Ontology in 2010: extensions and refinements. Nucleic Acids Research 38, D331–335 (2010). 19920128 10.1093/nar/gkp1018 PMC2808930

[b30] Zhao M., Chen Y., Qu D. & Qu H. TSdb: a database of transporter substrates linking metabolic pathways and transporter systems on a genome scale via their shared substrates. Sci China Life Sci 54, 60–64 (2011). 21253872 10.1007/s11427-010-4125-y

[b31] Karp P. D. . Expansion of the BioCyc collection of pathway/genome databases to 160 genomes. Nucleic Acids Res 33, 6083–6089 (2005). 16246909 10.1093/nar/gki892 PMC1266070

[b32] Kanehisa M. . KEGG for linking genomes to life and the environment. Nucleic Acids Res 36, D480–484 (2008). 18077471 10.1093/nar/gkm882 PMC2238879

[b33] Zhao M., Chen X., Gao G., Tao L. & Wei L. RLEdb: a database of rate-limiting enzymes and their regulation in human, rat, mouse, yeast and E. coli. Cell Res 19, 793–795 (2009). 19468287 10.1038/cr.2009.61

[b34] Thomas P. D. . PANTHER: a library of protein families and subfamilies indexed by function. Genome Res 13, 2129–2141 (2003). 12952881 10.1101/gr.772403 PMC403709

[b35] Schaefer C. F. . PID: the Pathway Interaction Database. Nucleic Acids Res 37, D674–679 (2009). 18832364 10.1093/nar/gkn653 PMC2686461

[b36] Zhao M. & Qu H. PathLocdb: a comprehensive database for the subcellular localization of metabolic pathways and its application to multiple localization analysis. BMC Genomics 11 Suppl 4, S13 (2010). 21143796 10.1186/1471-2164-11-S4-S13 PMC3005916

[b37] Croft D. . Reactome: a database of reactions, pathways and biological processes. Nucleic Acids Res 39, D691–697 (2011). 21067998 10.1093/nar/gkq1018 PMC3013646

[b38] Matthews L. . Reactome knowledgebase of human biological pathways and processes. Nucleic Acids Res 37, D619–622 (2009). 18981052 10.1093/nar/gkn863 PMC2686536

[b39] Becker K. G., Barnes K. C., Bright T. J. & Wang S. A. The genetic association database. Nat Genet 36, 431–432 (2004). 15118671 10.1038/ng0504-431

[b40] Kanehisa M., Goto S., Furumichi M., Tanabe M. & Hirakawa M. KEGG for representation and analysis of molecular networks involving diseases and drugs. Nucleic Acids Res 38, D355–360 (2010). 19880382 10.1093/nar/gkp896 PMC2808910

[b41] Osborne J. D. . Annotating the human genome with Disease Ontology. BMC Genomics 10 Suppl 1, S6 (2009). 19594883 10.1186/1471-2164-10-S1-S6 PMC2709267

[b42] Du P. . From disease ontology to disease-ontology lite: statistical methods to adapt a general-purpose ontology for the test of gene-ontology associations. Bioinformatics 25, i63–68 (2009). 19478018 10.1093/bioinformatics/btp193 PMC2687947

[b43] Hindorff L. A. . Potential etiologic and functional implications of genome-wide association loci for human diseases and traits. Proc Natl Acad Sci U S A 106, 9362–9367 (2009). 19474294 10.1073/pnas.0903103106 PMC2687147

[b44] Su A. I. . A gene atlas of the mouse and human protein-encoding transcriptomes. Proc Natl Acad Sci U S A 101, 6062–6067 (2004). 15075390 10.1073/pnas.0400782101 PMC395923

[b45] Forbes S. A. . COSMIC: mining complete cancer genomes in the Catalogue of Somatic Mutations in Cancer. Nucleic Acids Res 39, D945–950 (2011). 20952405 10.1093/nar/gkq929 PMC3013785

[b46] Aerts S. . Gene prioritization through genomic data fusion. Nat Biotechnol 24, 537–544 (2006). 16680138 10.1038/nbt1203

[b47] Gao J. . Integrative analysis of complex cancer genomics and clinical profiles using the cBioPortal. Sci Signal 6, pl1 (2013). 23550210 10.1126/scisignal.2004088 PMC4160307

[b48] Zhao M., Li X. & Qu H. EDdb: a web resource for eating disorder and its application to identify an extended adipocytokine signaling pathway related to eating disorder. Sci China Life Sci 56, 1086–1096 (2013). 24302289 10.1007/s11427-013-4573-2

[b49] Smoot M. E., Ono K., Ruscheinski J., Wang P. L. & Ideker T. Cytoscape 2.8: new features for data integration and network visualization. Bioinformatics 27, 431–432 (2011). 21149340 10.1093/bioinformatics/btq675 PMC3031041

[b50] Barabasi A. L. & Oltvai Z. N. Network biology: understanding the cell’s functional organization. Nat Rev Genet 5, 101–113 (2004). 14735121 10.1038/nrg1272

[b51] Xie C. . KOBAS 2.0: a web server for annotation and identification of enriched pathways and diseases. Nucleic Acids Res 39, W316–322 (2011). 21715386 10.1093/nar/gkr483 PMC3125809

